# Retraction: The helix of CO_2_, household income, and oil pricing under the assumption of Keynesian consumption function: A policy-mix scenario of oil-importing South Asia for SDGs-2030

**DOI:** 10.1371/journal.pone.0274232

**Published:** 2022-09-14

**Authors:** The PLOS ONE Editors

The *PLOS ONE* Editors retract this article [1] because it was identified as one of a series of submissions for which we have concerns about authorship, competing interests, and peer review. We regret that the issues were not addressed prior to the article’s publication.

SAA did not agree with the retraction. CG, HS, MUF, MN, FG and MNA either did not respond directly or could not be reached.
